# The Relationship Between Attitude Toward Pain and the Effects of Foam Rolling on Biomechanical Parameters of Soft Tissues Measured with the MyotonPRO Device

**DOI:** 10.3390/healthcare13212809

**Published:** 2025-11-05

**Authors:** Przemysław Dębski, Grzegorz Szlachta, Maciej Biały, Ewelina Białas, Kamil Kublin

**Affiliations:** 1 Institute of Physiotherapy and Health Sciences, The Academy of Physical Education, 40-065 Katowice, Poland; pdfizjoterapia@gmail.com (P.D.); mbfizjoterapia@gmail.com (M.B.); ebialas94@gmail.com (E.B.); 2 Physiotherapy Department, St. Luke’s Hospital, 43-309 Bielsko-Biala, Poland; kublinkamil@gmail.com

**Keywords:** foam rolling, pain catastrophizing, myotonometry, stiffness, pain attitude, physiotherapy, sports, pain

## Abstract

**Highlights:**

This study examined the relationship between pain-related attitudes and biomechanical changes in myofascial tissues following foam rolling. The results indicate that psychological factors, particularly pain catastrophizing, are correlated with FR-induced alterations.

**What are the main findings?**

- Pain-related attitudes are correlated with FR-induced changes
- Fewer associations were found after longer rolling, potentially indicating a time-dependent effect

**What is the implication of the main findings?**

- Identifying negative pain attitudes may improve pain management and enhance FR effectiveness
- Screening for pain beliefs may help tailor FR strategies

**Abstract:**

**Background:** Foam Rolling (FR) involves applying intense pressure to soft tissues, which can cause discomfort or pain due to the mechanical stimulation. This study aims to explore the relationship between individuals’ attitudes toward pain and the changes in the biomechanical properties of myofascial tissues induced by FR, as measured using myotonometry. Understanding this relationship may be valuable for optimizing recovery and performance in both recreational and athletic settings. **Methods:** Thirty-two healthy men (mean age: 24.3 ± 4.56 years) were randomly assigned to one of two groups: ROL30 (30 s of FR) and ROL120 (120 s of FR). The MyotonPRO device was used to evaluate changes in biomechanical parameters: stiffness, frequency, logarithmic decrement, relaxation time, and creep, before and after FR. Measurements were taken from the gastrocnemius, biceps femoris, erector spinae, and longissimus colli muscles. Data were analyzed for each muscle and as a combined sum across both sides of the body. Psychological factors were assessed using the Pain Catastrophizing Scale (PCS) and the Survey of Pain Attitudes (SOPA), analyzing both total and subscale scores. Correlations between PCS/SOPA scores and changes in myotonometric parameters were calculated using Spearman’s rank correlation coefficient. **Results:** In the ROL30 group, 11 significant correlations were found between myotonometric changes and PCS/SOPA scores (r ranging from −0.55 to −0.76 and from 0.54 to 0.77), with the most prominent correlation observed between the sum of decrement and PCS total score (r = −0.55). In the ROL120 group, 3 significant correlations were identified (r ranging from −0.60 to −0.62), including a notable one between the sum of decrement and PCS helplessness (r = −0.60). **Conclusions:** Attitudes toward pain appear to show associations with certain outcomes of foam rolling. These findings suggest that individual pain perceptions may be related to the applied force during FR and, consequently, the treatment’s effectiveness. Assessing pain-related attitudes beforehand could help personalize FR interventions, particularly in athletes and active individuals, where pain is a routine aspect of training. Identifying negative pain attitudes may also improve pain management and enhance FR effectiveness, though further research is needed. Future studies should include larger sample sizes and validated scales to better understand the role of pain attitudes and their potential causal influence on FR outcomes.

## 1. Introduction

Foam rolling (FR) is a tool-assisted self-massage technique targeting myofascial tissues and serves as a complementary approach to manual therapy. Evidence suggests that it contributes to short-term improvements in flexibility and recovery from exercise-induced muscle soreness, while also attenuating the sensitivity of latent myofascial trigger points [1]. The effect of rolling on the biomechanical parameters of myofascial tissue remains unclear. The discrepancy in the results obtained by the authors may be the result of many factors. Among them, the most frequently mentioned are: differences in parameters of FR such as time of rolling, huge variance between the anatomical measurement points, characteristics of participants, etc. [2].

FR is a method of applying intense pressure to soft tissues, often causing discomfort or sensations near the pain threshold. The changes observed following the application of FR appear to be unknown, yet likely of systemic origin. It has been shown to affect autonomic nervous system activity [3,4], and to increase the pressure pain threshold [5]. However, the latter finding has recently been questioned by more comprehensive evidence [6]. Notably, a painful stimulus is not required to induce changes in pain threshold [7]. It is therefore plausible that intense mechanoreceptor stimulation may trigger “pain inhibits pain” or other modulation mechanisms, with the magnitude of the stimulus determining the nature and extent of the physiological response.

Pain is often described as: “An unpleasant sensory and emotional experience associated with, or resembling that associated with, actual or potential tissue damage” [8]. Both the described phenomenon and intense discomfort below the pain threshold may play a role in differentiating the effects of therapy. For this reason, it seems reasonable to measure the approach to pain of people participating in studies using FR. There is a lack of research in the literature focusing on the impact of the emotional approach of respondents on the results of various types of intense mechanical therapy and this gap remains difficult to explain from the authors’ point of view. Mental load associated with high applied physical forces can differ among both patients and healthy subjects. It may affect muscle activity consciously or reflexively and may also lead some of the subjects to avoid intense stimulation. For this reason, it is important to consider assessing the approach to pain/discomfort in studies evaluating the impact of intensive manual or tool-based mechanical pressure therapies on the obtained results.

Pain catastrophization is often characterized as an exaggerated negative orientation toward noxious stimuli [9]. This variable appears to differentiate individuals across various aspects of society. Pain catastrophizing is related to pain and adjustment in borderline morbidly obese and morbidly obese patients with osteoarthritis. Patients engaging in high levels of pain catastrophizing reported higher levels of binge eating, lower eating self-efficacy and lower overall weight-related quality of life [10]. On the other side of scale in terms of unpleasant stimuli triathletes exhibited increased pain tolerance, decreased perceived pain, decreased fear of pain and pain catastrophizing than controls [11]. The authors suggest that such results may be “the chronic effect of extreme training, by unique inherent traits or by their combination”.

Fear-Avoidance Beliefs (FAB) as well as catastrophizing occur in this general population of non-patients [12] and has an impact on their movements [13]. Among manual therapy techniques, massage has been suggested to potentially influence FAB [14]; however, there is a lack of high-quality studies verifying such assumptions [15]. In the case of high-intensity techniques, it is worth asking whether the opposite relationship might exist, and whether a potential tendency to avoid intense pressure may be related to the outcomes of therapy, which served as the initial hypothesis for the study. For this reason, it is necessary to assess the relationship between the approach to pain and discomfort and the results obtained in high-intensity pressure therapies.

The aim of this study is to determine whether psychological factors, such as pain catastrophizing and attitude towards pain, are associated with the outcomes of foam rolling on the biomechanical properties of soft tissues assessed using myotonometry.

## 2. Materials and Methods

### 2.1. The Study Design

The enrollment to the study lasted from December 2019 to December 2021. The study was a single-blinded randomized, parallel-group, exploratory trial. The assessor who performed the measurements was blinded to both the participants’ group allocation and the intervention they received. The results of the experiment were described based on CONSORT 2025 Statement [16,17], SPIRIT 2013 Statement [18], TIDieR guide [19], and the statistical part was developed based on CHaMP [20].

### 2.2. Sample Size

**Figure 1:**
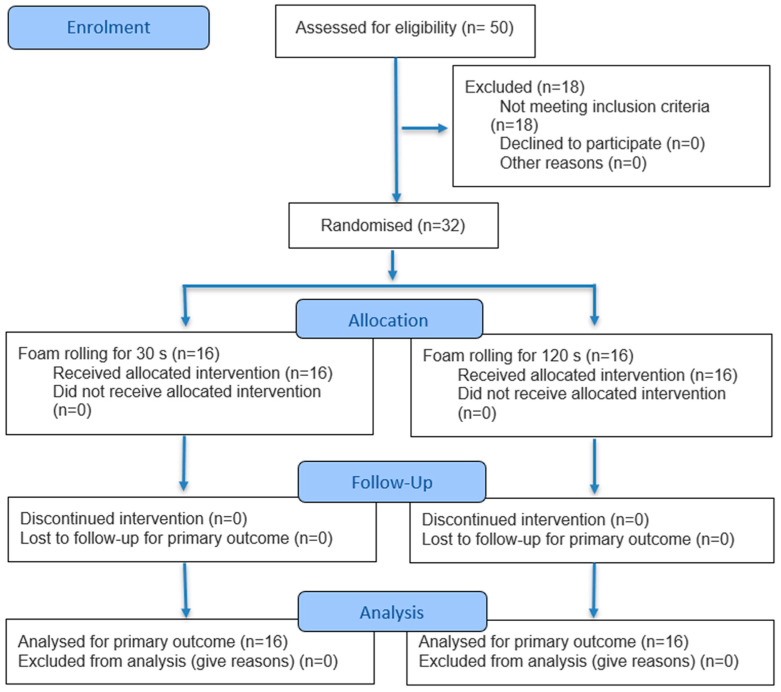
Flow diagram of the present study.

Out of 50 people from the general population, 32 subjects were selected for research using purposive sampling, and then randomly assigned to two groups, as presented in Figure 1. Randomization was performed using a draw of cards from an opaque container, ensuring both random sequence generation and allocation concealment. For a two-tailed correlation analysis (α = 0.05, *n* = 16 participants per group; two groups in total), the statistical power (1 − β) was 0.63 for an expected correlation of r = 0.55, and 0.82 for r = 0.65, as calculated using G*Power 3.1.9.2 program (Heinrich Heine University, Düsseldorf, Germany).

### 2.3. Participants

A total of 32 men aged 18–35 were recruited for the study to exclude changes in biomechanical parameters that could result from different developmental stages [21]. The main selection criteria were lack of generalized joint hypermobility measured in Beighton Hypermobility Scale, skinfold measurement test less than 3.5 cm, and medical history of pathologies including muscular and fascial tissues (orthopedics) as well as autoimmune, psychiatric and serious infectious diseases. The selection criterion of a skinfold thickness below 3.5 cm was based on the fact that the MyotonPRO device provides reliable measurements up to a subcutaneous fat tissue thickness of 2 cm. This threshold corresponds to approximately twice the permissible subcutaneous fat layer (as a skinfold consists of two layers) minus a 0.5 cm safety margin, as described in the study by Dębski et al. [2]. Medical history was verified with individual questionnaire.

### 2.4. Measures

MyotonPRO (Myoton AS, Tallin, Estonia) was used to examine the biomechanical parameters of tissues. Myotonometry represents a novel and non-invasive, easy-to-use method to characterize the biomechanical and viscoelastic muscle properties such as elasticity (logarithmic decrement—D), stiffness (S), tone (frequency—F), relaxation time (R) and creep (C) [22].

The measurement was performed three times at each of the designated points, using the triple measurement option (triplescan), before the intervention and within two minutes after the intervention. In the main examination, four points located along the posterior myofascial chain [23] were marked on the subject’s body in the prone position. This selection was made with the intention of assessing both local and remote associations of foam rolling. Such an approach is supported by the observations of Russo et al., who reported changes within the posterior myofascial chain following local application of self-myofascial release [24]. The GC (gastrocnemius) point was located in the upper third of the distance from the lower edge of the fibular head to the intersection of the central line of the Achilles tendon and the line connecting the lower edges of the malleoli. The BF (biceps femoris) point was positioned at half the distance between the upper edge of the fibular head and the lower edge of the ischial tuberosity. The LE (lumbar erector spinae) point was located within the erector spinae muscle at the lower third of the distance between the superior edge of the posterior superior iliac spine and the inferior edge of the external occipital protuberance, while the CE (cervical erector spinae) point was positioned at the upper third of this distance. All measurement points were located on the dominant side. Limb dominance was determined based on a battery of three tests: participant’s self-report, the chair climb test, and the ball kick test. A limb was classified as dominant when at least two of these tests indicated the same side as preferred.

The Pain Catastrophization Scale (PCS) and The Survey of Pain Attitudes (SOPA) were used to examine psychometric aspects of FR application.

The Pain Catastrophization Scale (PCS) is widely used, standard psychometric tool [25] self-assessment questionnaire to examine catastrophization among patients. It consists of 13 statements describing thoughts and feelings experienced during the pain. Each statement is scored on the 5-point scale. The PCS is divided into three subscales: rumination scale, magnification scale and helplessness scale. The scale presents good test–retest reliability of 0.88 (95% CI 5 0.83–0.93, range 0.73–0.97) and internal consistency of 0.92 (95% CI 5 0.91–0.93) [26].

The Survey of Pain Attitudes (SOPA) is one of the most commonly used questionnaires to assess pain-related beliefs. The SOPA-Brief (SOPA-B) contains 30 items that corresponds to seven domains of beliefs and attitudes towards pain: pain control, disability, harm, emotion, medication, solicitude and medical cure. The scale presents good internal consistency except the domain of medication [27,28].

### 2.5. Interventions

Participants were randomly assigned to two groups (*n* = 16 each). The first group performed hamstring foam rolling for 30 s (ROL30), while the second group performed the same intervention for 120 s (ROL120). In both groups, the procedure was applied to the dominant extremity.

While seated with legs extended, the participant supported himself with his hands on the floor and, upon instruction, shifted his body weight onto a foam roller positioned under the hamstring muscles. He then rolled his body smoothly along the length of the muscles, from the ischial tuberosity to the popliteal fossa, and back. The rolling was performed using a firm, smooth Physioroll^®^ roller (dimensions: 31 × 16 cm, diameter: 15 cm), compliant with ISO 9001:2009 [29] and ISO 14001:2005 [30] quality standards. To control the tempo of movement, all interventions were performed with the use of a metronome set at 60 beats per minute. The direct force applied to the roller was not measured; however, in this position, the pressure forces are generally reported to range from approximately 25% to 55% of body mass, depending on roller placement [31]. Participants were instructed to apply as much pressure as possible, which is a standard procedure in this type of study [32].

### 2.6. Statistical Analysis

The analysis included results from two psychometric tools: the PCS (rumination, magnification, helplessness subscales, total score) and the SOPA (emotionality, control, physical harm subscales, total score). The assessment of foam rolling effects on the biomechanical properties of soft tissues was based on the difference between the averaged values of three MyotonPRO measurements (parameters: F, D, S, C, R) performed in Triple Scan mode before and after the intervention (difference = pre-rolling − post-rolling). Measurements were taken at the following points: GC, BF, LE, CE and the overall effect was also expressed as the sum of the values across these points. To simplify the description, abbreviations were introduced (e.g., CE-F refers to parameter F at point CE).

The normality of the variable distribution was assessed using the Shapiro–Wilk test. Group differences in anthropometric characteristics were assessed using Student’s *t*-test. The Mann–Whitney U test was used to assess differences in PCS and SOPA scores, as well as in biomechanical parameters, between groups. Spearman’s rank correlation coefficient was used to assess the relationship between PCS and SOPA scores and changes in the biomechanical parameters of soft tissues. The interpretation of the r-Spearman correlation coefficient was in accordance with Schober’s et al. (2018) proposition: <0.1 negligible, 0.10–0.39 weak, 0.40–0.69 moderate, 0.70–0.89 strong, ≥0.90 very strong correlation [33]. The critical level of statistical significance was set at *p* < 0.05. The Statistica 13.3 software (TIBCO Software Inc., Palo Alto, CA, USA) was used to compute all calculations.

## 3. Results

### 3.1. Baseline Characteristics

Baseline characteristics of the study groups, including age, body height, body mass, and body mass index (BMI), are presented in Table 1. Statistical analysis showed no significant differences between groups for any of these variables (*p* > 0.05).

In the comparison of psychometric scale results and differences in biomechanical parameters of soft tissues between groups, significantly higher PCS rumination (7 vs. 6.5; *p* = 0.03) and total PCS scores (12 vs. 10.5; *p* = 0.03) were observed in the ROL30 group compared to the ROL120 group. Full results are presented in Table 2.

### 3.2. Correlations

#### 3.2.1. Analysis of Correlations in the ROL30 Group

Analysis of correlations between psychometric variables and myotonometric changes identified 11 significant associations within the ROL30 group.

One of correlations concerned PCS and the myotonometric parameter F, specifically: a strong negative correlation between PCS magnification and LE-F (r = −0.76).

Five significant moderate negative correlations were identified between PCS scores and the myotonometric parameter D. These were: PCS rumination vs. CE-D (r = −0.68), PCS rumination vs. SUM D (r = −0.55), PCS magnification vs. SUM D (r = −0.61), PCS sum vs. CE-D (r = −0.65), and PCS sum vs. SUM D (r = −0.55).

Additionally, two significant moderate positive correlations were observed between SOPA physical harm and the myotonometric parameter D, in the following order: SOPA physical harm vs. BF-D (r = 0.54) and vs. LE-D (r = 0.65).

One significant moderate negative correlation was identified between PCS helplessness and the myotonometric parameter S; this was: PCS helplessness vs. LE-S (r = −0.62).

One strong positive correlation was also observed between PCS magnification and each of the following myotonometric parameters: LE-R (r = 0.77) and LE-C (r = 0.77). Table 3 presents detailed correlations between the parameters described above.

#### 3.2.2. Analysis of Correlations in the ROL120 Group

In the ROL120 group, three significant moderate negative correlations were observed between PCS and the myotonometric parameter D. These were: PCS magnification vs. GC-D (r = −0.62), PCS helplessness vs. LE-D (r = −0.61), and PCS helplessness vs. SUM-D (r = −0.60). Detailed data are presented in Table 4.

## 4. Discussion

The aim of this study was to examine the association between psychological factors (including pain catastrophizing and pain-related attitudes) and the effects of FR on soft tissue biomechanical properties measured using myotonometry. The findings suggest that individuals’ pain-related beliefs and attitudes may be linked to the magnitude of changes in tissue biomechanics following foam rolling.

In the context of PCS questionnaire scores, the most relevant and meaningful findings of the present study revealed that, in the ROL30 group, five significant negative correlations were observed between changes in the D parameter and PCS scores. The sum of the D parameter showed moderate negative correlations with the PCS total score (r = −0.55), as well as with the PCS Rumination subscale (r = −0.55) and PCS Magnification subscale (r = −0.61). Additionally, significant negative correlations were observed between the CE-D and both the PCS total score and the PCS Rumination subscale (r = −0.65 and −0.68, respectively).

In the ROL120 group, only 3 significant correlations were observed, all of which concerned the D parameter and PCS. These were PCS helplessness subscale and the sum of D and LE-D (r = −0.60 and −0.61, respectively), as well as PCS magnification subscale and GC-D (r = −0.62).

This may suggest that individuals who tend to catastrophize pain in terms of both magnification, rumination, and feelings of helplessness exhibit smaller changes in tissue elasticity due to a smaller change in the D parameter. Such effects may be partly due to the avoidance of applying strong pressure on the roller (in individuals with high levels of catastrophizing), possibly in fear of experiencing greater discomfort or even pain. A higher incidence of statistically significant correlations was observed in the ROL30 group compared to the ROL120 group. This difference may be attributed to significantly higher baseline levels of rumination in the PCS subscale (7 vs. 6.5; *p* = 0.03), as well as in the total PCS score (12 vs. 10.5; *p* = 0.03) in the ROL30 group.

Another notable finding in the ROL30 group is a significant correlation between PCS Magnification and nearly all changes in biomechanical parameters within the LE muscle, except for D. Specifically, significant correlations were observed between PCS Magnification and the following parameters: F (r = −0.76), S (r = −0.62), R (r = 0.77), and C (r = 0.77). Although these findings are of interest, they remain difficult to interpret.

In the context of the SOPA, moderate positive correlations were also observed between SOPA Physical Harm and BF-D and LE-D (0.54 and 0.65, respectively) in the ROL30 group. However, no significant correlation was found between SOPA Physical Harm and the sum of D parameters in this group.

In the ROL120 group, none of these correlations were statistically significant. This suggests that stronger beliefs as reflected in the SOPA Physical Harm subscale, which includes the belief that pain signals tissue damage and that physical activity or exercise might be harmful—are associated with greater changes in the D parameter, indicating more pronounced alterations in tissue elasticity. Although these findings appear to contrast with the correlations observed between PCS scores and the D parameter, it is noteworthy that the one of these correlations occurred in a muscle directly subjected to foam rolling.

In summary, the group that applied FR for 30 s exhibited a greater number of correlations compared to the group that performed FR for 120 s. While previous literature has not demonstrated a significant impact of rolling duration on therapeutic outcomes, these findings suggest that duration may constitute a more influential factor in specific populations. Based on the current data, the number of correlations between approach to pain factors and the amount of change in biomechanical parameters was lower following the longer FR duration. This may indicate that extended mechanical stimulation yields effects that are less influenced by cognitive-emotional components, thereby contributing to a more homogeneous response within the study population. It is plausible that, analogous to massage, FR could contribute to the reduction in fear-avoidance beliefs (FAB), as theoretically proposed by Hunt et al. [14]. It should be mentioned once more that the force application in the present study was not objectively quantified, and therefore this hypothesis is speculative in nature. On the other hand, compensatory unloading would not necessarily correspond to a reduction in applied force; rather, it could simply involve moving the roller to a less painful area, accompanied by a disruption of movement linearity or tempo. For this reason, it is unclear whether any potential compensations could be measured by assessing the applied force of the roller.

Interestingly, only one significant correlation was found between the rolled muscle (BF-D) and psychological factors (SOPA Physical Harm in the ROL30 group) in relation to PCS and SOPA scores. The predominance of correlations in the LE and with the total D parameter suggests a global or proximal pattern of correlation, potentially driven by intersegmental compensation in participants exhibiting higher levels of pain catastrophizing. Observations regarding the effects of self-myofascial release on the contralateral side of the body [34] as well as along the posterior myofascial chain [24] have already been reported. However, this hypothesis requires further investigation.

There are many studies describing the effect of FR on the Pressure Pain Threshold (PPT), Delayed Onset Muscle Soreness (DOMS), Exercise Induced Muscle Damage (EIMD) scale [5], or visual analog scale (VAS) [35,36] as well as autonomic responses mentioned earlier in text. Analysis of the results of the mentioned parameters can lead to interesting conclusions. In one such study, autonomic nervous system parameters such as Heart Rate Variability (HRV) did not change after FR application. On the contrary, the initial level of this parameter determined the level of pain experienced during the application [37].

A natural consequence of similar information is an attempt to study the co-occurrence of autonomic arousal (and therefore perceived pain), but also beliefs, attitudes toward perceived pain and the effects of FR therapy. In the case of FR therapy, these factors can be variables that determine the applied compression force, which in turn can affect the results obtained.

To date, no studies have been identified that directly investigate this specific topic. However, several previous works have examined the influence of mechanical compression force on treatment outcomes. Aboodarda et al. [38] observed greater improvements in range of motion (ROM) with more intense stimulation. However, because the authors compared FR with massage, it is difficult to draw definitive conclusions regarding the specific effects of compression force.

Another relevant study is that of Grabow et al., which did not find compression intensity—measured as a percentage of the Rating of Perceived Pain (RPP)—to significantly affect variables such as strength, jump performance, or ROM [39]. However, the most relevant finding in the context of the present study comes from their pilot data, in which the authors noted that “the average force that a person would exert while rolling a muscle was strongly dependent on the day and the individual.” Similar observation served as a conceptual basis for further investigation into the role of pain perception in the present study and is considered a critical yet frequently overlooked factor in the current FR literature.

Finally, it is worth noting the findings of Hirose et al. [40], who reported no significant influence of FR pressure or perceived pain intensity on ROM gains. However, their study involved only 20 participants, limiting the generalizability of the results. Importantly, none of the studies mentioned above included variables that directly assessed the individual’s approach to pain, nor did they examine biomechanical parameters. This represents a significant gap in the literature and underscores the novelty and importance of the current study.

### Limitations

The authors wish to emphasize the main limitation of the study, which concerns the study sample that included only men aged 18–35 years. Although these criteria limit the generalizability of the findings to a broader population, they allowed for the selection of a homogeneous group. This decision was primarily motivated by potential differences related to age and sex, including variations in adipose tissue levels, menstrual cycle–related factors in women, and possible differences in pain perception. The present study should be extended to a larger and more diverse population.

## 5. Conclusions

To some extent, the approach to pain is correlated with the effects of FR, which may account for the reduced benefits observed in certain subjects. A longer duration of foam rolling may reduce the influence of pain-related psychological factors on biomechanical outcomes, thereby enhancing the consistency of responses within the study population. Additionally, pain assessment scales should be incorporated into future research on FR and other forms of intense mechanical therapy, such as sports massage or other intensive fascial interventions. Examining attitudes toward pain in individuals undergoing FR may enhance the effectiveness of this form of self-therapy in certain populations.

## Figures and Tables

**Table 1:** Descriptive statistics (mean ± standard deviation) and *t*-test comparisons of anthropometric characteristics between groups.

Characteristics	ROL30 (*n* = 16)	ROL120 (*n* = 16)	*p*
Age (years)	24.19 ± 3.89	24.41 ± 5.23	0.26
Height (cm)	175.63 ± 5.45	178.65 ± 8.18	0.37
Weight (kg)	75.81 ± 8.36	77.41 ± 8.18	0.93
BMI (kg/m^2^)	24.54 ± 1.89	24.26 ± 2.21	0.55

**Table 2:** Descriptive statistics and results of between-group comparisons for subscales and total scores of the Pain Catastrophizing Scale (PCS) and the Survey of Pain Attitudes (SOPA), as well as for changes (pre–post) in soft tissue biomechanical parameters in the groups performing foam rolling for 30 s (ROL30) and 120 s (ROL120). Results are presented as medians with the (first; third quartile).

Parameters	ROL30 (*n* = 16)	ROL120 (*n* = 16)	*p*
PCS rumin.	7.00 (4.50; 8.50)	6.50 (3.50; 7.50)	0.03 *
PCS magnif.	3.00 (2.00; 4.00)	3.00(2.00; 4.00)	0.21
PCS helples.	2.50 (1.00; 3.50)	2.00 (1.00; 4.50)	0.36
PCS sum	12.00 (10.00; 16.00)	10.50 (8.00; 14.50)	0.03 *
SOPA emot.	0.88 (0.13; 1.75)	1.50 (0.25; 1.63)	0.81
SOPA contr.	2.10 (1.80; 2.90)	2.50 (1.90; 2.80)	0.16
SOPA phys. Harm.	2.70 (2.20; 3.00)	2.30 (2.00; 2.60)	0.29
SOPA Sum	15.48 (12.20; 19.90)	13.88 (10.83; 19.13)	0.98
GC-F	5.00 (4.38; 5.65)	4.93 (4.42; 5.67)	0.37
BF-F	5.05 (4.79; 5.38)	5.16 (4.65; 5.70)	0.64
LE-F	4.62 (4.38; 4.93)	4.88 (4.55; 5.16)	0.31
CE-F	5.32 (4.91; 5.92)	5.36 (4.98; 5.78)	0.50
Sum-F	19.77 (19.16; 21.07)	20.24 (19.65; 21.38)	0.14
GC-D	−5.46 (−5.94; −4.87)	−5.13 (−5.55; −4.71)	0.82
BF-D	−5.14 (−5.23; −4.77)	−5.13 (−5.53; −4.58)	0.91
LE-D	−4.65 (−4.88; −4.34)	−4.90 (−5.19; −4.74)	0.58
CE-D	−5.46 (−5.97; −5.09)	−5.33 (−5.60; −5.06)	0.65
Sum-D	−20.46 (−21.45; −19.66)	−20.36 (−21.36; −19.56)	0.90
GC-S	99.25 (92.04; 111.17)	97.96 (91.58; 104.82)	0.51
BF-S	95.88 (90.41; 106.08)	100.25 (90.69; 113.73	0.21
LE-S	90.02 (79.95; 99.64)	91.43 (83.55; 99.16)	0.07
CE-S	97.37 (94.71; 115.45)	102.26 (92.63; 112.96)	0.49
Sum-S	391.83 (362.82; 418.17)	386.99 (374.99; 428.50)	0.11
GC-R	−95.90 (−110.15; −87.52)	−90.63 (−97.30; −85.68)	0.41
BF-R	−93.58 (−99.72; −85.65)	−96.48 (−105.67; −87.32)	0.60
LE-R	−88.55 (−93.87; −73.45)	−91.68 (−96.08; −84.50)	0.55
CE-R	−102.05 (−116.78; −90.55)	−98.02 (−104.83; −91.85)	0.75
Sum-R	−370.82 (−410.03; −350.53)	−375.25 (−399.20; −360.58)	0.51
GC-C	−5.51 (−6.13; −4.98)	−5.96 (−6.41; −5.57)	0.39
BF-C	−5.68 (−5.93; −5.21)	−5.49 (−6.07; −5.11)	0.84
LE-C	−5.77 (−6.38; −5.48)	−5.43 (−5.78; −5.19)	0.90
CE-C	−5.05 (−5.45; −4.51)	−5.12 (−5.35; −4.83)	0.95
Sum-C	−22.30 (−23.00; −20.90)	−22.20 (−23.02; −20.81)	0.81

*—statistically significant (*p* < 0.05), GC—gastrocnemius, BF—biceps femoris, LE—Lumbar erector spinae, CE—cervical erector spinae, F—frequency, D—logarithmic decrement, S—stiffness, R—relaxation time, C—creep.

**Table 3:** Correlations between the subscales and total scores of the Pain Catastrophizing Scale (PCS) and the Survey of Pain Attitudes (SOPA) and the changes (pre–post) in soft tissue biomechanical parameters in the ROL30 group (30 s of foam rolling; *n* = 16).

	PCS Rumin.	PCS Magnif.	PCS Helplesn.	PCS Sum	SOPA Emotions	SOPA Control	SOPA Phys. Harm.	SOPA Sum
GC-F	0.00	−0.01	−0.18	−0.06	−0.04	−0.23	0.21	0.09
BF-F	0.05	0.12	−0.41	−0.09	−0.40	−0.23	−0.24	−0.34
LE-F	−0.46	−0.76 *	0.02	−0.46	−0.14	0.49	0.18	0.16
CE-F	−0.03	−0.27	0.34	−0.01	−0.02	0.08	−0.01	0.03
Sum-F	−0.09	−0.15	0.05	−0.11	−0.23	−0.22	−0.03	−0.14
GC-D	−0.04	−0.43	0.08	−0.07	−0.21	−0.04	0.07	−0.02
BF-D	−0.02	−0.01	0.02	−0.04	0.05	0.17	0.54 *	0.32
LE-D	0.22	−0.06	0.24	0.23	0.30	0.28	0.65 *	0.49
CE-D	−0.68 *	−0.46	−0.09	−0.65 *	−0.43	−0.26	−0.16	−0.39
Sum-D	−0.55 *	−0.61 *	0.01	−0.55 *	−0.31	−0.02	0.19	−0.04
GC-S	−0.21	−0.03	−0.32	−0.26	−0.23	0.04	0.04	0.01
BF-S	0.17	0.18	−0.30	0.05	−0.17	−0.16	−0.04	−0.12
LE-S	−0.35	−0.62 *	−0.10	−0.38	−0.06	0.44	0.30	0.24
CE-S	−0.24	−0.46	0.36	−0.19	−0.05	0.05	−0.01	−0.01
Sum-S	−0.39	−0.28	−0.09	−0.40	−0.22	−0.07	−0.08	−0.12
GC-R	0.08	0.02	0.46	0.23	0.27	0.23	−0.00	0.14
BF-R	−0.07	−0.08	0.28	0.04	0.37	0.36	0.32	0.41
LE-R	0.47	0.77 *	0.01	0.48	0.16	−0.41	−0.29	−0.19
CE-R	0.10	0.36	−0.43	0.05	0.02	−0.13	−0.04	−0.05
Sum-R	0.30	0.42	0.05	0.37	0.41	0.08	−0.04	0.15
GC-C	−0.06	−0.10	0.45	0.10	0.11	0.23	−0.05	0.06
BF-C	−0.21	−0.17	0.22	−0.09	0.30	0.32	0.35	0.38
LE-C	0.44	0.77 *	−0.07	0.45	0.08	−0.42	−0.24	−0.19
CE-C	0.09	0.38	−0.45	0.04	0.00	−0.08	0.00	−0.02
Sum-C	0.27	0.42	0.10	0.39	0.30	0.06	0.05	0.16

*—statistically significant (*p* < 0.05), GC—gastrocnemius, BF—biceps femoris, LE—Lumbar erector spinae, CE—cervical erector spinae, F—frequency, D—logarithmic decrement, S—stiffness, R—relaxation time, C—creep.

**Table 4:** Correlations between the subscales and total scores of the Pain Catastrophizing Scale (PCS) and the Survey of Pain Attitudes (SOPA) and the changes (pre–post) in soft tissue biomechanical parameters in the ROL120 group (120 s of foam rolling; *n* = 16).

	PCS Rumin.	PCS Magnif.	PCS Helplesn.	PCS Sum	SOPA Emotions	SOPA Control	SOPA Phys. Harm	SOPA Sum
GC-F	−0.22	−0.17	0.08	−0.15	−0.10	−0.15	0.12	−0.10
BF-F	−0.14	0.03	−0.11	−0.13	−0.09	−0.38	−0.01	−0.12
LE-F	0.06	−0.19	−0.23	−0.00	0.02	−0.09	0.13	0.00
CE-F	−0.11	0.28	0.00	−0.10	−0.16	−0.15	−0.06	−0.26
Sum-F	−0.02	0.26	0.12	0.07	−0.27	−0.43	−0.05	−0.31
GC-D	−0.04	−0.62 *	−0.46	−0.32	0.30	−0.16	0.26	0.18
BF-D	0.03	0.11	−0.06	0.09	0.22	−0.27	0.41	0.25
LE-D	−0.19	−0.05	−0.61 *	−0.37	0.34	0.05	−0.07	0.23
CE-D	0.16	0.07	−0.12	0.09	0.16	−0.02	0.01	0.22
Sum-D	0.11	−0.21	−0.60 *	−0.16	0.48	−0.27	0.32	0.41
GC-S	−0.02	−0.22	0.04	−0.03	0.10	−0.18	0.17	0.07
BF-S	−0.05	0.02	−0.09	0.02	−0.15	−0.17	0.09	−0.10
LE-S	−0.08	−0.19	−0.30	−0.13	0.10	−0.11	0.17	0.05
CE-S	−0.16	0.10	−0.09	−0.22	0.02	−0.09	−0.06	−0.13
Sum-S	−0.14	0.06	−0.09	−0.09	0.10	−0.17	0.20	0.04
GC-R	0.19	0.08	0.00	0.15	−0.05	0.19	−0.10	−0.00
BF-R	−0.00	−0.20	−0.12	−0.15	0.20	0.10	−0.02	0.10
LE-R	0.16	0.25	0.29	0.20	−0.09	−0.02	−0.14	−0.05
CE-R	0.20	−0.11	−0.01	0.21	0.00	0.00	0.03	0.13
Sum-R	0.07	−0.07	−0.14	−0.03	−0.06	0.02	−0.18	−0.09
GC-C	0.20	0.07	0.02	0.15	−0.05	0.23	−0.13	0.00
BF-C	−0.07	−0.24	−0.17	−0.22	0.29	0.09	0.07	0.17
LE-C	0.19	0.39	0.31	0.26	−0.07	0.03	−0.14	−0.01
CE-C	0.15	0.04	0.02	0.22	0.06	0.06	0.06	0.21
Sum-C	0.08	0.08	−0.02	0.05	0.09	0.11	−0.08	0.08

*—statistically significant (*p* < 0.05), GC—gastrocnemius, BF—biceps femoris, LE—Lumbar erector spinae, CE—cervical erector spinae, F—frequency, D—logarithmic decrement, S—stiffness, R—relaxation time, C—creep.

## Data Availability

The data presented in this study are available on request from the corresponding author due to legal reasons.

## Abbreviations

The following abbreviations are used in this manuscript:

FR	Foam Rolling
FAB	Fear-Avoidance Beliefs
GC	Gastrocnemius muscle
BF	Biceps Femoris muscle
LE	Lumbar Erector spinae muscle
CE	Cervical Erector spinae muscle
PCS	Pain Catastrophization Scale
SOPA	The Survey of Pain Attitudes
SOPA-B	SOPA-Brief
ROL30	The first group performed hamstring foam rolling for 30 s
ROL120	The second group performed the same intervention for 120 s
D	Elasticity (logarithmic decrement)
R	Relaxation time
S	Stiffness
F	Tone (frequency)
C	Creep
BMI	Body Mass Index
SUM D	overall effect on parameter D
